# Rapid adsorption of benzotriazole onto oxidized carbon cloth as an easily separable adsorbent

**DOI:** 10.1038/s41598-023-44067-w

**Published:** 2023-10-09

**Authors:** Emad K. Radwan, Rehab A. Omar, Ahmed S. Moursy

**Affiliations:** Water Pollution Research Department, National Research Centre, 33 El Buhouth St, Dokki, Giza, 12622 Egypt

**Keywords:** Environmental chemistry, Analytical chemistry

## Abstract

A commercial carbon cloth (CC) was oxidized by HNO_3_ acid and the features of the plain and oxidized CC were evaluated. The results of characterization illustrated that HNO_3_ oxidization duplicated the oxygen-containing functional groups and the surface area of the CC. The adsorption performance of the plain and oxidized CC (Oxi-CC) toward benzotriazole (BTR) was compared. The results disclosed that the uptake of BTR by oxidized CC was greater than the plain CC. Thence, the affinity of oxidized CC toward BTR was assessed at different conditions. It was found that the adsorption was quick, occurred at pH 9 and improved by adding NaCl or CaCl_2_ to the BTR solution. The kinetic and isotherm studies revealed that the surface of Oxi-CC is heterogeneous and the adsorption of BTR follows a physical process and forms multilayer over the Oxi-CC surface. The regenerability and reusability study illustrated that only deionized water can completely regenerate the Oxi-CC and that the Oxi-CC can be reused for five cycles without any loss of performance. The high maximum adsorption capacity of Dubinin–Radushkevich isotherm model (252 mg/g), ease of separation and regeneration, and maintaining the adsorption capacity for several cycles revealed the high efficiency and economical and environmental feasibility of Oxi-CC as an adsorbent for BTR.

## Introduction

The worldwide rapid industrial progress has introduced numerous synthetic chemicals in humans’ everyday life. The inefficient treatment of wastewater leads to the contamination of surface and ground water with several contaminants of emerging concerns (CECs). These CECs have deleterious effects on the environment, live beings and the natural balance of biomes^1–5^.

Benzotriazole (BTR, C_6_H_5_N_3_) is one of the CECs that is widely used as corrosion inhibitor, antifreeze, anti-fog, anti-rust, drug precursor, cooling and hydraulic fluid, UV absorber, and dishwashing detergent. Therefore, it is produced in large quantities. BTR is characterized by high water solubility (28 g/L) and bio-recalcitrance which limits its removal by the conventional water/wastewater treatment processes. The wide applications range, mass production and limited removal efficiency lead to the widespread detection of BTR in the aquatic environments. However, the high toxicity, ability to disrupt the endocrine system and long-term negative effects of BTR raised the public concerns towards its presence in the aquatic environments^4,6–9^. As a result, it is critical to use cutting-edge treatment methods to get rid of BTR from aquatic environments.

Advanced oxidation process^10^, membrane filtration^11^, biological processes^12^, combined processes^13^ have been applied to remove CECs from water/wastewater. However, amongst the different advanced treatment processes, adsorption has a well-established efficiency for the removal of hazardous contaminants in addition to its easy operation, economic feasibility, low-energy consumption, and insensitivity to toxic substances. Consequently, adsorption became the most widely applied^4,7,14–18^. So far, among the many materials that have been utilized as adsorbents, carbonaceous materials stand out because of their demonstrated affinity for various contaminants^19,20^. Activated carbon has three forms; granular, powdered and fibers, felts or cloth^16,18^. Unlike carbon cloth (CC), granular (GAC) and powdered activated carbons (PAC) suffer from the release of fine particles and dusts, and also, suffer from significant headloss due to their small grain size, and the accumulation of solid debris at the filter surface^17,19^.

Recently, CC has attracted significant consideration in water treatment because of its several advantages. Comparative to GAC and PAC, CC has superior mechanical and structural integrity, less diffusion limitations, ease of handling, straightforward recovery post-treatment, smaller reactor size, rapid adsorption kinetics and effectiveness similar to or even better than GAC and PAC^14,16–18,20–22^. In spite of these prominent merits, the application of CC for water treatment still limited, relative to GAC and PAC, till now^23^. Researchers are currently devoting efforts to reduce the cost of CC production, endow selectivity and specificity to a definite contaminant, evaluate its efficiency in multicomponent systems, among other topics^24,25^. However, enhancing the adsorption effectiveness of CC is one of the key challenges that could promote its practical application.

Most previous studies used plain commercial carbon cloth for the adsorption of organic contaminants^16,18–20,22^. Very few studies considered the modification of CC. For instance, Zulfiqar et al.^3^ prepared a porous activated carbon cloth modified by a silane polymer and applied it for the separation of oil/water mixtures. Generally, the pre-treatment processes of adsorbents aim to improve its adsorption performance via adding functional groups, increasing the surface area or both. Surface oxidation is an approach that introduces oxygen-containing functional groups on the material’s surface. These oxygen-containing functional groups are potential adsorption sites and increase the material’s hydrophilicity and wetting properties^26^.

To the best of our knowledge, the adsorption of the contaminant of emerging concern benzotriazole by oxidized carbon cloth has not been reported yet. Therefore, this study targets filling this literature gap. For the first time, this study aims at evaluating and improving the adsorption efficiency of a commercially available carbon cloth toward the adsorption of benzotriazole. The approach of introduction of new oxygen-containing functional groups on the surface of the commercially available carbon cloth to improve its adsorption efficiency was pursued in this study. A simple nitric acid hydrothermal process was followed to oxidize the commercial carbon cloth and introduce new oxygen-containing surface functional groups. Then, the chemical and textural properties of the plain and oxidized carbon cloth were finely characterized. A series of batch adsorption experiments were executed with the aim of evaluating the effect of nitric acid oxidation on the adsorption efficiency, finding the optimum conditions of the adsorption process, understanding the effect of ionic strength on the adsorption, testing the regeneration and reusability of the oxidized carbon cloth, and understanding the adsorption mechanism. Different kinetics and isotherm models were applied to describe the mechanism of adsorption.

## Materials and methods

### Materials

Carbon cloth (CC, ELAT-hydrophilic plain cloth) was purchased from fuel cell store. Nitric acid (HNO_3_, 69%), and benzotriazole (99%) were purchased from Sigma-Aldrich.

### Nitric acid hydrothermal oxidation

A piece (4 × 6 cm) of carbon cloth was immersed in 40 mL HNO_3_ (69%) contained in a 50 mL Teflon-lined stainless autoclave. The autoclave was sealed and maintained at 90 °C for 16 h. After cooling to room temperature naturally, the oxidized carbon cloth (Oxi-CC) was taken out and washed with copious amount of deionized water then dried.

### Plain and oxidized carbon cloth characterization

The morphology and elemental composition of the plain and oxidized carbon cloth were investigated by field emission scanning electron microscopy (FESEM, TESCAN VEGA 3) and energy dispersive X-ray analysis (EDX, Bruker), respectively. Before analysis, the samples were coated by gold using Quorum Q 150 ES (UK) magnetron sputtering machine. The functional groups were identified using attenuated total reflection Fourier transform infrared (ATR-FTIR, Jasco 4100). The N_2_ adsorption–desorption isotherms at 77 K was obtained using BELSORP-max surface analyzer. The samples were outgassed at 150 °C overnight before analysis. The specific surface area (S_BET_) was calculated according to Brunauer–Emmett–Teller and the pore size distribution was determined by nonlocal density functional theory (NLDFT) from the N_2_ adsorption isotherm. The diameter of the carbon fibers was determined manually using ImageJ 1.54b software. The point of zero charge was determined according to the salt addition method^27^.

### Evaluation of adsorption performance

Before performing the adsorption experiments, a series of working solutions of BTR were prepared by proper dilution of 100 mg/L stock solution. All solutions were prepared using deionized water obtained from Milli-Q water purification system. The UV spectra of the working solutions were recorded using a double-beam UV–Vis spectrophotometer (JASCO V730, Japan) and are shown in Fig. S1a. As given by the Figure, the absorption maximum is located at 273 nm. A nine-point standard curve was established by plotting the absorbance at 273 nm *vs.* concentration (Fig. S1b). A straight line with high coefficient of determination (R^2^ = 0.999) was obtained over the concentration range 0–100 mg/L. Therefore, this standard curve was used to convert the absorbance of unknown samples to concentration.

The adsorption experiments were conducted in batch mode using an orbital shaker incubator (DAIHAN ThermoStable™ IS-30, Korea) at 200 rpm and 26 °C. In a typical experiment, precisely weighed pieces of carbon cloth were added in Erlenmeyer flasks containing BTR solution. After a determined time, the concentration of BTR was determined and the amount of BTR adsorbed per gram of carbon cloth (q, mg/g) and removal percentage (R%) were calculated by Eqs. (1) and (2), respectively:

1 $${\text{q}}_{\text{t}} \, \text{ } = \, \, \left({\text{C}}_{\text{o}} - \, {\text{C}}_{\text{t}}\right) \frac{\text{V}}{{\text{w}}},$$

2 $$\text{R (\%) =}\left(1-\frac{{\text{C}}_{\text{t}} }{{\text{C}}_{\text{o}}}\right)\times \text{ 100}\text{,}$$

wherein C_o_ and C_t_ (mg/L) are the concentrations of BTR before the beginning of the experiment and after contact with the carbon cloth for t (min), respectively, V (L) is the volume of BTR solution, and *w* (g) is the mass of the carbon cloth piece.

The effects of initial pH (pH_o_) of BTR solution, ionic strength, adsorption time, initial concentration of BTR solution and reusability of the carbon cloth were evaluated using the aforementioned procedure. In the pH_o_ effect study, the pH_o_ of 50 mL BTR solution (20 mg/L) was adjusted to 3, 5, 7, and 9 then contacted with 50 mg of carbon cloth. Samples were collected periodically and the concentrations of BTR were measured. In the ionic strength effect study, a specific amount of the NaCl or CaCl_2_ (25–300 mg/L) was added to 50 mL BTR solution (20 mg/L) pre-adjusted to the optimum pH_o_ then the BTR solution was contacted with 50 mg of carbon cloth. In the initial concentration effect study, 50 mg of carbon cloth was added to a series of 50 mL BTR solutions of different initial concentrations (5–45 mg/L). After contacting for 2 h, the remaining concentrations of BTR were measured. In the reusability study, the exhausted carbon cloth was regenerated by shaking with deionized water for 1 h before reusing for another adsorption cycle. The regeneration efficiency was calculated according to Eqs. (S4) and (S5). Each adsorption experiment was performed three times under the same conditions. The reported results are the average of the triplicate analysis.

The obtained adsorption kinetic and isotherm data were treated with the nonlinear forms of the common adsorption mathematical models in order to explore the adsorption mechanism. The tested kinetic and isotherm models are given in details in the supporting information. The validity of the tested kinetic and isotherm models was evaluated based on several error functions as discussed in the supporting information. OriginPro 2021 software was used for graphing, and analysis of the data.

## Results and discussions

### Characteristics of the plain and oxidized carbon cloth

**Figure 1:**
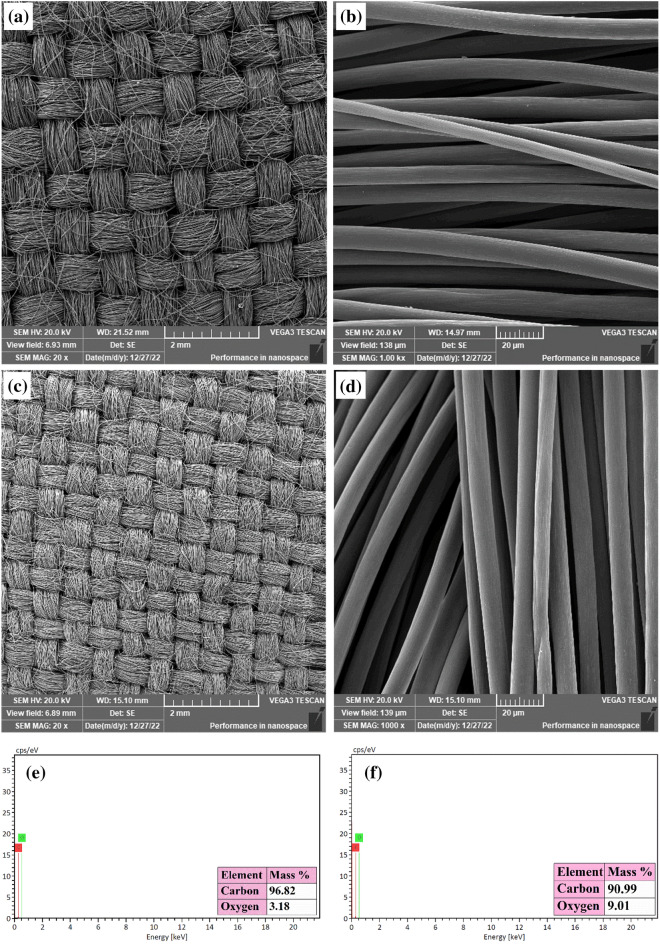
SEM micrographs and EDX spectra of plain (**a,b,e**) and oxidized (**c,d,f**) carbon cloth.

With the aim of increasing the potential adsorption site, a commercially available plain carbon cloth was treated with nitric acid. The nitric acid treatment results in the oxidation of carbon atoms on the surface of carbon cloth where the C–C and/or the C–H bonds are oxidized to C–O bonds. Thus, nitric acid treatment increases the oxygen-containing functional groups such as –C=O, –C–O, and –COOR^28^ which are potential adsorption sites. The morphology of the plain and oxidized carbon cloth is displayed in Fig. 1. The plain carbon cloth (Fig. 1a) is made out of many carbon fibers that are gathered to form very similar woven braids. The individual carbon fibers have a smooth surface that is free of pores, defects and grooves. The diameters of the carbon fibers were determined from the high magnification image (Fig. 1b) and found to range between 5.5 and 9.7 µm.

The SEM images of the oxidized carbon cloth displayed in Fig. 1c,d indicate that the oxidation process has not changed the structure and morphology of the carbon cloth. Cheng et al.^28^ and Moloudi et al.^29^ have reported similar observation before.

The elemental composition of the carbon cloth was determined before and after the oxidation process by EDX analysis. The EDX spectra of both plain and oxidized carbon cloth (Fig. 1e,f, respectively) show the presence of the peaks of oxygen and carbon elements. The inset tables of Fig. 1e,f show the mass percentage of carbon and oxygen. It can be seen that the mass percentage of oxygen in the oxidized carbon sample is 2.8 times that of the plain carbon cloth. This result indicates increasing the content of oxygen-containing functional groups in the oxidized carbon cloth which implies the success of the oxidation process.

**Figure 2:**
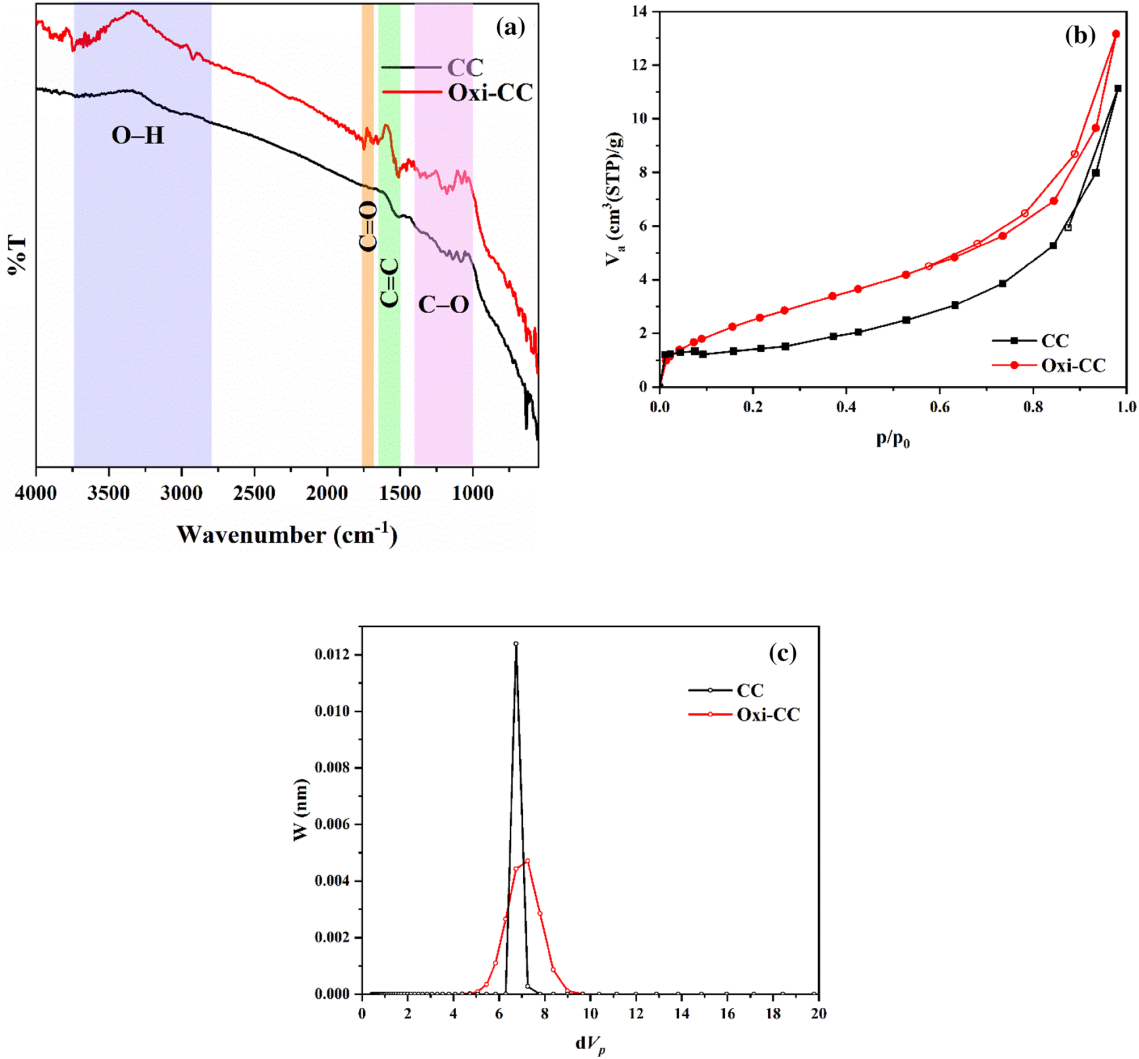
FTIR spectra (**a**), N_2_ adsorption–desorption isotherms (**b**), and NLDFT pore size distribution (**c**) of plain (CC) and oxidized (Oxi-CC) carbon cloth.

The surface functional groups of the plain and oxidized carbon cloth were assessed using FTIR analysis and the spectra are presented in Fig. 2a. The IR spectrum of the plain CC revealed the presence of aliphatic and aromatic hydrocarbons in addition to few oxygen-containing functional groups. Specifically, the weak broad peaks around 3700 cm^−1^ and 3040 cm^−1^ are assignable to the stretching vibration of O–H and =C–H of aliphatic or aromatic hydrocarbons, respectively^3^. The peak at 1519 cm^−1^ is for aromatic C=C bending^3,30^. The peak at 1460 cm^−1^ results from the –C–H bending vibration of CH_3_ and CH_2_ groups. The peaks in the range 1000–1400 cm^−1^ can be ascribed to the stretching vibration of the C–O bond^29^. The band at around 610 cm^−1^ is due to the C–H bond of the –C≡C–H.

Clear differences between the spectra of the plain and oxidized carbon cloth can be noticed. Altogether, the intensifying and appearance of additional peaks related to the oxygen-containing functional groups and the receding of the intensity of the peaks related to the carbon atoms can be observed in the spectrum of the oxidized carbon cloth. Specifically, the intensity of the peaks at 3700 cm^−1^ (O–H stretching), and at the range 1000–1400 cm^−1^ (C–O stretching) was increased. Additional prominent peaks related to the stretching vibration of O–H, C=O (of carboxylic acids amides, ketones, aldehydes, lactone, or esters), and aromatic C=C groups appeared at 2916 cm^−1^, 1740–1685 cm^−1^^30^, and 1540 cm^−1^, respectively. The appearance of these peaks proves the oxidation of plain carbon cloth. In addition, it can be noted that the intensity of the band for the C–H bond of the –C≡C–H decreased after the oxidation process which indicates the oxidation of the C–H bonds. All in all, the FT-IR results give solid evidence for the successful oxidation and increasing the oxygen-containing functional groups of the carbon cloth. This result is in accordance with earlier studies that found carbon cloth’s content of oxygen-containing functional groups increased as a result of oxidation^29–33^.

The surface area and porous structure of a material is an important property which affect its adsorption efficiency. Therefore, the N_2_ adsorption–desorption isotherms of the plain and oxidized carbon cloth were measured and used to determine their surface area, pore volume, pore size and pore size distribution. Figure 2b shows the obtained N_2_ adsorption–desorption isotherms.

The first step in the interpretation of physisorption isotherm is to define the type of the isotherm^34^. The type of the isotherm identifies the nature of the adsorption process. The International Union of Pure and Applied Chemistry (IUPAC) categories physisorption isotherms into six groups^34,35^. Herein, the physisorption isotherms of both the plain and oxidized carbon cloth presented in Fig. 4a are concave to the *p/p*_0_ axis at low *p/p*_0_ and convex afterward. This shape matches well with the reversible type II isotherm which characterizes the nonporous or microporous materials and implies unrestricted mono-/multi-layer adsorption. In addition, the presence of hysteresis loops in the physisorption isotherms of plain and oxidized carbon cloth is depicted in Fig. 2b. The observation that the hysteresis loops have the lower limit of desorption branches at the cavitation-induced *p/p*_*0*_ indicates that these loops are of the H3 type.

The Brunauer, Emmett and Teller (BET) method was applied to determine the specific surface area (S_BET_) and total pore volume (V_tot_) of the plain and oxidized carbon cloth. The calculated S_BET_ and V_tot_ of plain carbon cloth were 5.2 m^2^/g and 0.017 cm^3^/g, respectively, while those of oxidized carbon cloth were 10.2 m^2^/g and 0.020 cm^3^/g, respectively. These results indicate that the oxidation of the plain carbon cloth increased the surface area and total pore volume significantly which agrees with literature. Previously, Vautard et al.^36^, Gao and Zhao^37^, and Pittman et al.^38^ reported that oxidation of carbon fibers by nitric acid increases the surface area and oxygen-containing functional groups.

The pore size distribution of the plain and oxidized carbon cloth was determined from the N_2_ adsorption isotherm by the non-local density functional theory (NDLFT) model. Figure 2c shows the obtained pores size distribution curves. Both the plain and oxidized carbon cloth is mainly mesoporous with a mean pore diameter of 6.7 nm and 7.2 nm, respectively. Moreover, the pore size distribution curve of the oxidized carbon cloth is relatively wider than that of the plain carbon cloth. This might be due to widening the distance between the carbon fibers by the nitric acid during the oxidation process.

Joining the observed morphology (Fig. 1), physisorption isotherm type (Fig. 2b), and the pore size distribution curves (Fig. 2c) indicates that the fibers of both plain and oxidized carbon cloth are nonporous and the distances between the fibers form mesopores.

### Adsorption of benzotriazole

**Figure 3:**
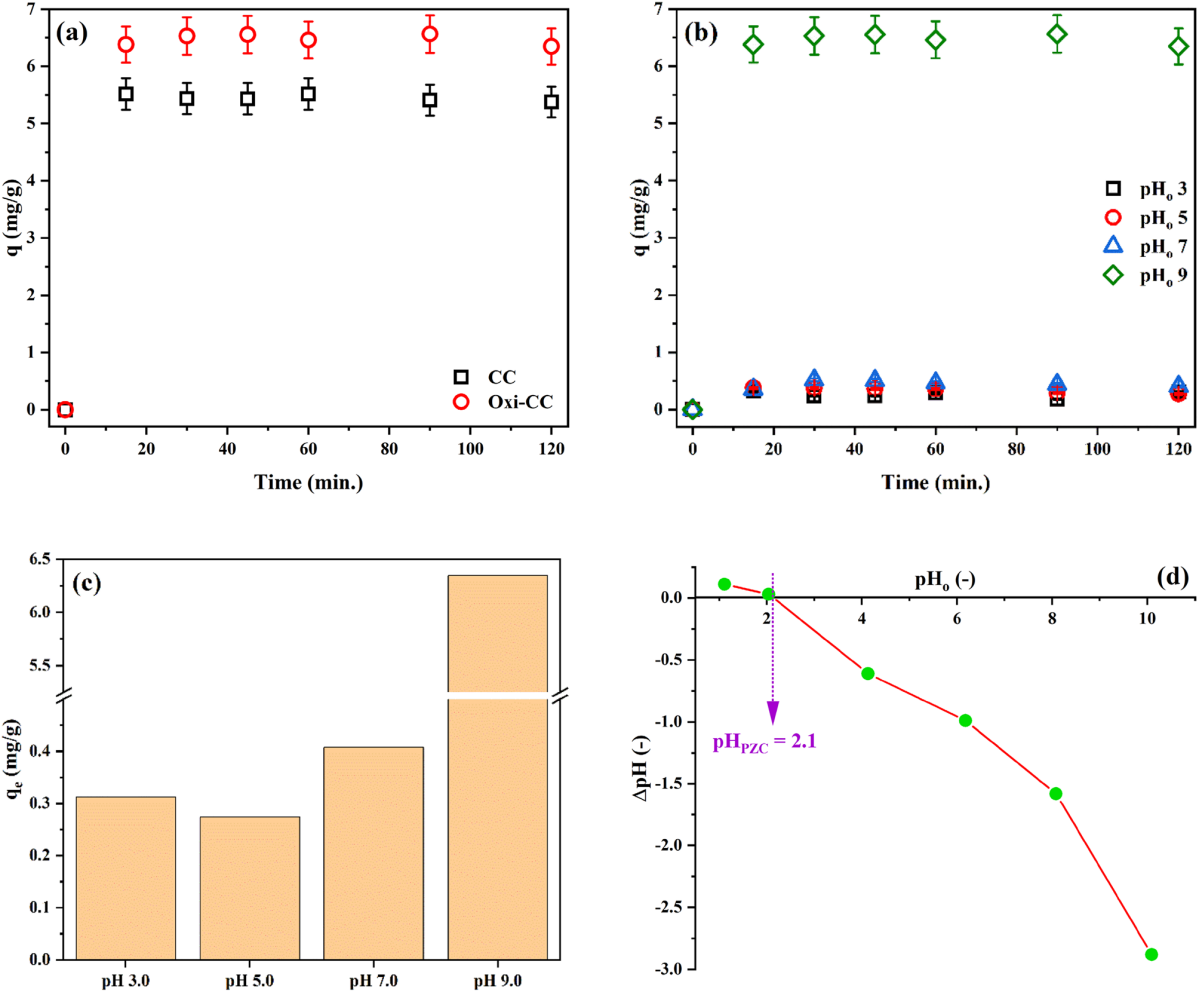
(**a**) Removal of BTR by CC and Oxi-CC, (**b**,**c**) effect of pH_o_ on BTR removal by Oxi-CC, and (**d**) pH_PZC_ of Oxi-CC.

The characteristics of carbon cloth such as lightweight, compactness, high hydraulic conductivity, low mass transfer resistances, and ease of arrange in a variety of stable configurations simplify and encourage its practical application^19,25^. The effectiveness of the plain and oxidized carbon cloth for the adsorption of benzotriazole from aqueous solutions was tested. This experiment was performed using 1 g/L of CC or Oxi-CC, and 20 mg/L BTR solution adjusted to pH_o_ 9. Figure 3 presents the results. It is abundantly clear that the removal of BTR by both CC and Oxi-CC was quite fast. The amount of adsorbed BTR increased sharply after the first contact with either CC or Oxi-CC then remained unchanged thereafter. Previously, Xu et al.^4^ reported a similar pattern for the adsorption of BTR by Zn–Al–O binary metal oxide where the amount of adsorbed BTR increased steeply to 0.26 mg/g after the first contact then remained constant. In general, it is recognized that the adsorption kinetic by CC is fast due to the small diameters of its consisting fibres^25^. Notably, the amount of BTR adsorbed by the Oxi-CC (6.53 mg/g) was considerably higher than that adsorbed by the CC (5.51 mg/g). This observation signifies the role of oxidation process in enhancing the adsorption performance of carbon cloth. Thus, the mechanism and factors affecting the adsorption of BTR by Oxi-CC was further explored.

The solution pH affects the surface charges of the adsorbent and ionization of the adsorptive. Thus, it mostly plays a critical role in the adsorption process. Figure 3b depicts the data obtained for the adsorption of BTR by 1 g/L of Oxi-CC at different pH_o_ as a function of contact time. The results show that Oxi-CC can adsorb 6.81 mg/g of BTR at pH_o_ 9, while at pH_o_ ranging from 3 to 7 relatively little adsorption takes place.

**Scheme 1:**
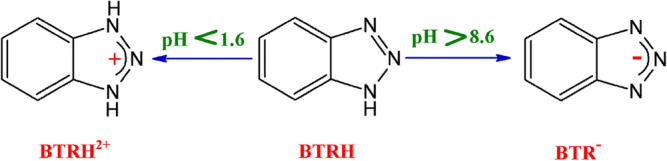
Forms of benzotriazole at different pH values.

The surface charge of the Oxi-CC was investigated at different pH values in order to explain the results of pH effect study. The difference between the initial and final pH vs. the initial pH is plotted in Fig. 3c. The figure shows that the surface of Oxi-CC has a pH_PZC_ of 2.1. Thus, the surface of Oxi-CC has a net electronegative charge under the studied pH range. On the other side, BTR behaves as a weak base (pK_a_ = 1.6) and a weak acid (pK_a_ = 8.6)^4,6,9^, therefore, it has three forms in aqueous solutions as displayed in Scheme 1. It exists in the protonated form (BTRH^2+^) at pH < 1.6, molecular (unionized) form at pH between 1.6 and 8.6, and deprotonated form (BTR^−^) at pH > 8.6. Thus, under the experimental conditions, BTR exist in the molecular form in the pH_o_ range 3–7 and the deprotonated form at pH_o_ 9.

**Scheme 2:**
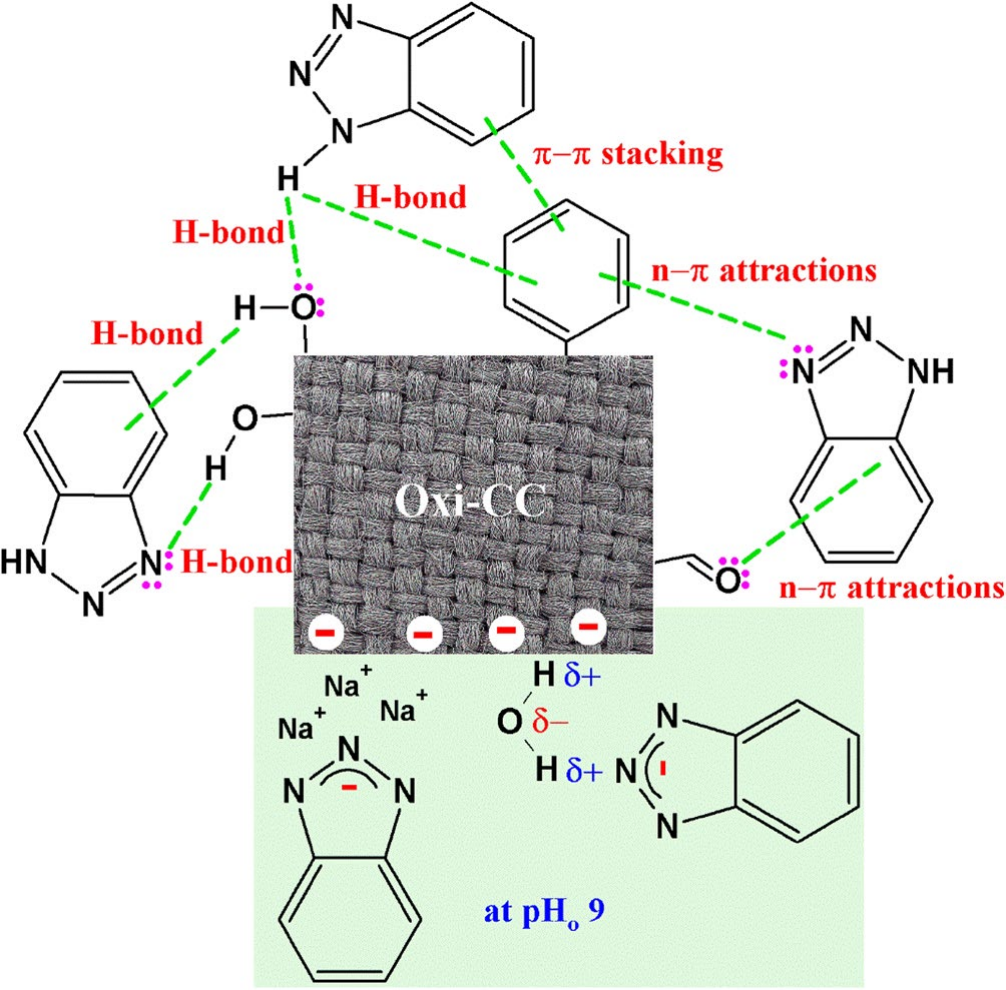
Some probable interactions between Oxi-CC and BTR.

In general, the adsorption of aromatic compounds can be driven by one or more of electrostatic attractions, ion exchange, hydrogen-bond interactions, hydrophobic interactions, Van der Waals’ interactions, dipole–dipole interactions, and π–π stacking^4,39^. As illustrated above, at pH_o_ 3–7, BTR exists in the molecular form and the Oxi-CC has a net negative charge. So, electrostatic attraction mechanism can be excluded. The FTIR of the Oxi-CC revealed the presence of aliphatic and aromatic hydrocarbons as well as oxygen-containing functional groups. Therefore, in this study, the possible interactions between the BTR and the Oxi-CC, presented in Scheme 2, are (a) π–π stacking between the π electrons of the aromatic rings of BTR and the Oxi-CC, (b) hydrogen-bond interactions between (i) the hydrogen of the –NH group and of BTR and either the benzene rings (Yoshida hydrogen-bond) or the oxygen containing functional groups of the Oxi-CC (dipole–dipole hydrogen-bond), and (ii) the hydrogen of the –OH groups on the surface of Oxi-CC and the benzene ring of BTR or the triazole nitrogen atoms, and (c) n-π electron donor–acceptor interaction between (i) the lone electron pair of the triazole nitrogen atoms and the aromatic rings on the Oxi-CC surface and (ii) the lone electron pair of the carbonyl groups of the Oxi-CC and the benzene ring of BTR. Beforehand, Hu et al.^9^ reported that hydrogen bonding drives the adsorption of BTR onto Zn-Al layered double oxide. Likewise, Hasanzadeh et al.^40^ and Sarker et al.^41^ reported that a combination of hydrophobic interactions, π–π stacking and electrostatic interactions underlies the adsorption of BTR onto modified magnetic biochar and Co-based metal azolate framework, respectively.

It is known that pH has insignificant effect on the hydrophobic and π–π interactions^40,41^. So, relatively slight change in the amount adsorbed can be observed in the pH_o_ range 3–7. At pH_o_ 9, electrostatic repulsion between the deprotonated form of BTR and the negative charges on the surface of Oxi-CC is predicted to dominates. Surprisingly, a considerably increase in the amount adsorbed can be observed at pH_o_ 9. Similarly, Hu et al.^9^ reported that Zn-Al layered double oxides adsorb a significant amount of BTA at pH > 8, despite the predicted repulsive forces between the Zn-Al layered double oxides and BTR, and attributed this observation to the involvement of a mechanism other than electrostatic interactions in the adsorption process without further clarifications.

A possible explanation of the observed increase in adsorption efficiency at pH_o_ 9 is the involvement of like-charge attraction mechanism. In general, the mechanism of like-charge attraction still ambiguous, however, bridging water, bridging counter-ions, and counter-ions bonded to only one of the like-charge ions are commonly debated as the underlying forces for the like-charge attraction^42^. Monovalent counter-ions can bound to the like-charge species hence screen the charge and diminish the electrostatic repulsion. But to generate attraction, non-electrostatic attraction must be involved. On the other side, multivalent counter-ions can invert the charge by bounding to one of the like-charged species and/or act as a bridge by bounding to both like-charge species. Thus, drives the like-charged attraction without the need for other attraction mechanisms^42–44^. In the present study there are two probable reasons for the observed like-charged attraction. First, the Na^+^ ions introduced to the medium during the adjustment of pH_o_ weaken the repulsive forces between the negatively charged Oxi-CC and the deprotonated form of BTR by the screening effect of Na^+^ ions^44^ (Scheme 2). Such weakening of the repulsive forces lets the hydrophobic and π–π interactions take place and drive the adsorption process. Second, the polar water molecules can mediate the attraction between the like-charged Oxi-CC and BTR. The slightly positive charge on hydrogen atoms of the water molecules can act as a bridge between the like-charged Oxi-CC and deprotonated BTR (Scheme 2). It has been reported previously that water molecules can invert the electrostatic repulsion into electrostatic attraction^45^. In both cases, the suggested like-charge adsorption mechanism highpoints the significance of non-electrostatic forces^46^.

To elucidate the involvement of hydrophobic attractions, like-charge attraction mechanism and the important role of cations on the adsorption of BTR onto Oxi-CC, the effect of both monovalent and divalent cations was investigated. Na^+^ and Ca^2+^ were used as representatives for monovalent and divalent cations, respectively. The results showed that the addition of 25 mg/L of either NaCl or CaCl_2_, increased the value of q_e_ by 15% (7.85 mg/g) or 16% (7.92 mg/g), respectively. However, the value of q_e_ insignificantly changed by further addition of NaCl and gradually increased by further addition of CaCl_2_ reaching 8.14 mg/g (20% increment) at 300 mg/L CaCl_2_. These results prove that the presence of monovalent or divalent cations diminishes the predicted electrostatic repulsion between the anionic BTR and the negatively charged Oxi-CC (at pH_o_ 9) and enables and increases the hydrophobic attractions. The slightly higher improvement of adsorption efficiency in case of Ca^2+^ suggests that it might contribute in the adsorption by act as bridge via bounding to both deprotonated BTR and negatively charged Oxi-CC. It is widely reported that increasing the ionic strength causes an increase in the hydrophobic attractions and decreases the electrostatic interactions^47,48^.

**Figure 4:**
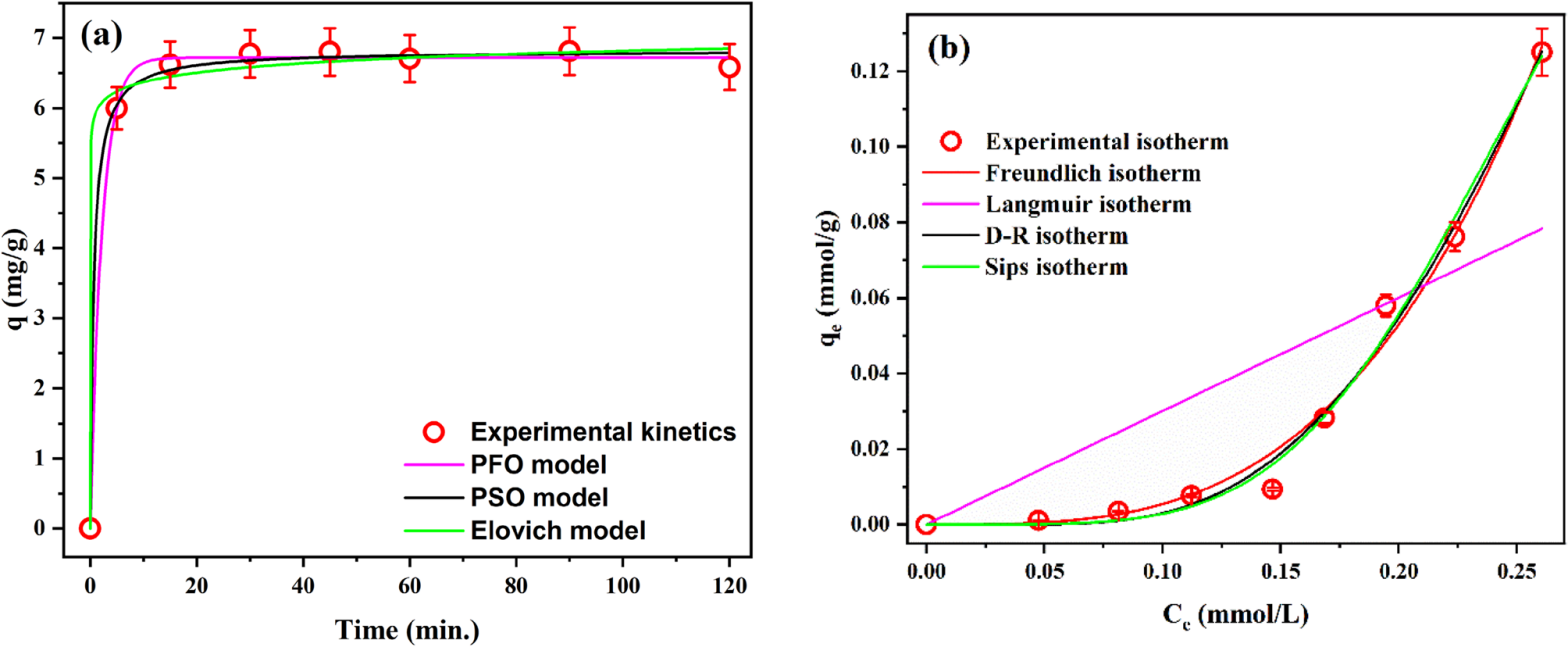
Experimental and models’ fitted curves of the adsorption (**a**) kinetics and (**b**) isotherm.

The adsorption process was performed as a function of contact time to determine the equilibrium time. It is obvious from Fig. 4a that the adsorption of BTR onto the Oxi-CC is instantaneous where the equilibrium state was only attained after 15 min. This fast adsorption can be ascribed to the abundance of adsorption sites and the large concentration gradient at the start of the adsorption process which promoted the migration of BTR from the aqueous to the solid phase and have reduced the mass transfer^8^. This fast adsorption suggests that the adsorption of BTR by the Oxi-CC occurs thru external surface adsorption^4^. Noteworthy that relative to previous studies on BTR adsorption, the equilibration time in this study was considerably short. For example, Hu et al.^9^ reported that the equilibrium state for the adsorption of BTR onto Zn-Al layered double oxide was 160 min. Xu et al.^4^ reported an equilibrium time of 30 min for the adsorption of BTR by Zn–Al–O binary metal oxide adsorbent. Furthermore, the amount of BTR adsorbed at equilibrium by the Oxi-CC (6.81 mg/g) was considerably higher than several other adsorbents in the literature. Yu et al.^8^ reported that the adsorption capacity of polyvinyl chloride microplastics toward BTR at equilibrium was 0.0626 mg/g. Xu et al.^4^ obtained an equilibrium adsorption capacity of 0.26 mg/g for BTR using Zn–Al–O binary metal oxide. Hongling et al.^7^ obtained a maximum equilibrium adsorption capacity of 1.28 mg/g for BTR adsorption using Ca-montmorillonite (Ca-Mt). However, there are some other reported powdered adsorbents that have higher equilibrium adsorption capacity than Oxi-CC. For example, Hongling et al.^7^ reported a maximum equilibrium adsorption capacity of 18.89 mg/g and 13.86 mg/g for propylbis (dodecyldimethyl) ammonium chloride and propylbis (octadecyldimethyl) ammonium chloride modified Ca-Mt, respectively. Undoubtedly, powdered adsorbents normally have higher adsorption capacity than immobilized ones, so comparing the efficiency of powdered adsorbents to Oxi-CC isn’t logical.

To further investigate the kinetics of adsorption and get some details about the adsorption mechanism, the experimental kinetic data was fitted to the pseudo-first-order (PFO)^49^, pseudo-second-order (PSO)^50^, and Elovich^51^ models. The fit curves are depicted in Fig. 4a and the resulting parameters besides the coefficient of determination (R^2^), chi-square (χ^2^), and root mean square error (RMSE) are provided in Table 1.

**Table 1:** Parameters of the applied kinetics and isotherm models.

Kinetic models		Isotherm models	
**PFO**		**Freundlich**	
R^2^	0.9988	R^2^	0.9872
χ^2^	0.008	χ^2^	0.00003
RMSE	0.09	RMSE	0.0053
k_1_	0.45 ± 0.03	K_F_	10.49 ± 3.54
q_e_	6.72 ± 0.04	n_F_	0.30 ± 0.02
**PSO**		**Langmuir**	
R^2^	0.9983	R^2^	0.6528
χ^2^	0.011	χ^2^	0.00075
RMSE	0.11	RMSE	0.0274
k_2_	0.23 ± 0.04	K_L_	2.83 × 10^–4^ ± 3.68
q_e_	6.82 ± 0.05	q_L_	1.06 × 10^3^ ± 1.38 × 10^7^
**Elovich**		**D–R**	
R^2^	0.9945	R^2^	0.9901
χ^2^	0.036	χ^2^	0.00002
RMSE	0.19	RMSE	0.0046
*α*	3.70 × 10^12^ ± 4.43 × 10^13^	K_D–R_	0.19 ± 0.01
*β*	5.16 ± 1.87	q_D–R_	2.12 ± 0.45
		**Sips**	
		R^2^	0.9906
		χ^2^	0.00002
		RMSE	0.0049
		K_S_	486.97 ± 1093.54
		n_S_	4.63 ± 1.04
		q_S_	0.25 ± 0.12

The values of the R^2^, χ^2^, and RSME obtained for the different kinetic models were compared in order to determine the suitability of the models to the experimental kinetic data and the most compatible model. The values of R^2^ were higher than 0.99 for all of the tested kinetic models which indicate that the three models can predict the experimental data. This observation implies that a sophisticated mechanism triggered the adsorption process. Noticeably, the value of R^2^ was slightly higher for the PFO and the values of χ^2^, and RSME were the lowest among the studied models. Therefore, it can be concluded that the kinetics of BTR adsorption onto Oxi-CC is more fitted to the PFO model. Several previous studies found that the PFO gives the better fit for their kinetic data^20,52^. The PFO model is based on a direct proportion between the rate of adsorption and the difference in equilibrium concentration and the amount adsorbed with time^52^. Also, the PFO is common for adsorption systems in which adsorption occur via diffusion thru the interface and physisorption is the rate determining step^53–55^.

Adsorption isotherm describes the relation between the concentration of adsorptive in the liquid and solid phases. It is essential for the design of adsorption process and to understand the interactions between the adsorptive and adsorbent. In this study, the adsorption isotherm was determined in batch mode at 26 °C using 1 g/L of the Oxi-CC and different initial concentrations (5–45 mg/L) of BTR solution adjusted to pH_o_ 9. The obtained experimental adsorption isotherm displayed in Fig. 4b shows that the amount adsorbed of BTR increases with increasing the initial concentration of BTR solution. The experimental adsorption isotherm belongs to the S-type sub-group 1 of Giles classification^56^. The S-type sub-group 1 isotherm indicates that the initial adsorption of BTR facilitates the adsorption of more BTR and high initial concentration of BTR enhances the adsorption. Also, it indicates that the adsorbed BTR is vertically oriented on the surface of Oxi-CC and surface saturation has not been attained. Xu et al.^4^ reported S-type isotherm for the adsorption of BTR onto Zn–Al–O binary metal oxide.

The non-linear forms of four isotherm models, namely, Langmuir^57^, Freundlich^58^, Dubinin–Radushkevich (D–R)^59^, and Sips^60^ were used to analyze the experimental adsorption isotherm. The regression plots of the applied isotherm models are shown in Fig. 4b and the calculated values of both models’ parameters and goodness-of-fit functions are listed in Table 1. Visual examination of Fig. 4b shows that among the applied adsorption isotherms, Langmuir model cannot fit the experimental adsorption isotherm data. This observation is supported by the calculated values of R^2^. Freundlich, D–R, and Sips models have R^2^ values greater than 0.98 while Langmuir has R^2^ of 0.65. Thus, the R^2^ values indicate the good fit of the Freundlich, D–R, and Sips models and the poor fit of Langmuir model. Moreover, the values of the goodness-of-fit indicators are consistent with this conclusion where very minor differences of χ^2^, and RSME for Freundlich, D–R, and Sips models can be observed. The common character of the Freundlich, D–R, and Sips models is that they can describe multilayer adsorption onto a heterogeneous surface. Each of these models provides additional information about the nature of the adsorption process. Freundlich model assumes interactions between adsorbates and increasing the amount adsorbed with increasing the adsorptive initial concentration^58^. The D–R model assumes a key role of Van der Waal’s forces in the formation of multilayer and enables the determination of whether the adsorption is a physical or a chemical process^59^. It is argued that the adsorption is dominated by physical process when the mean adsorption free energy $\left(E=\frac{1}{{\left(2{K}_{D{-}R}\right)}^{0.5}}\right)$ is less than 8 kJ/mol and chemical process when E is greater than 8 kJ/mol^55,61^. In this work, the calculated value of E is 1.62 kJ/mol. Therefore, the adsorption of BTR onto the Oxi-CC is mainly physical process. This inference is consistent with the findings of the kinetic study, which indicated that the rate-determining step is physisorption. The D–R model also provides valuable data about the maximum adsorption capacity of a material (the parameter *q*_*D–R*_). The calculated value of *q*_*D–R*_ in this study is 2.12 ± 0.45 mmol/g (252.33 ± 53.54 mg/g). This value is 235 times than what Xu et al.^4^ reported (1.07 mg/g), demonstrating the high efficiency of the Oxi-CC for the removal of BTR. Regrettably, the value of *q*_*D–R*_ for removing BTR using other adsorbents has not been found in the literature. The Sips exponent (n_s_) is commonly used to derive the heterogeneity factor (m_S_ = 1/n_S_) which defines the heterogeneity of the adsorbent surface. The value of m_S_ ranges between 0 and 1, an m_S_ of 1 indicates a homogeneous surface while a value less than 1 indicates a heterogeneous surface^62,63^. In this work, the calculated m_S_ is 0.26 revealing the heterogeneous nature of the surface of Oxi-CC. To sum up, the isotherm study indicated that the Oxi-CC has a heterogeneous surface with high adsorption affinity toward BTR adsorption. And that BTR adsorbs onto Oxi-CC via a physical process and forms multilayer.

**Figure 5:**
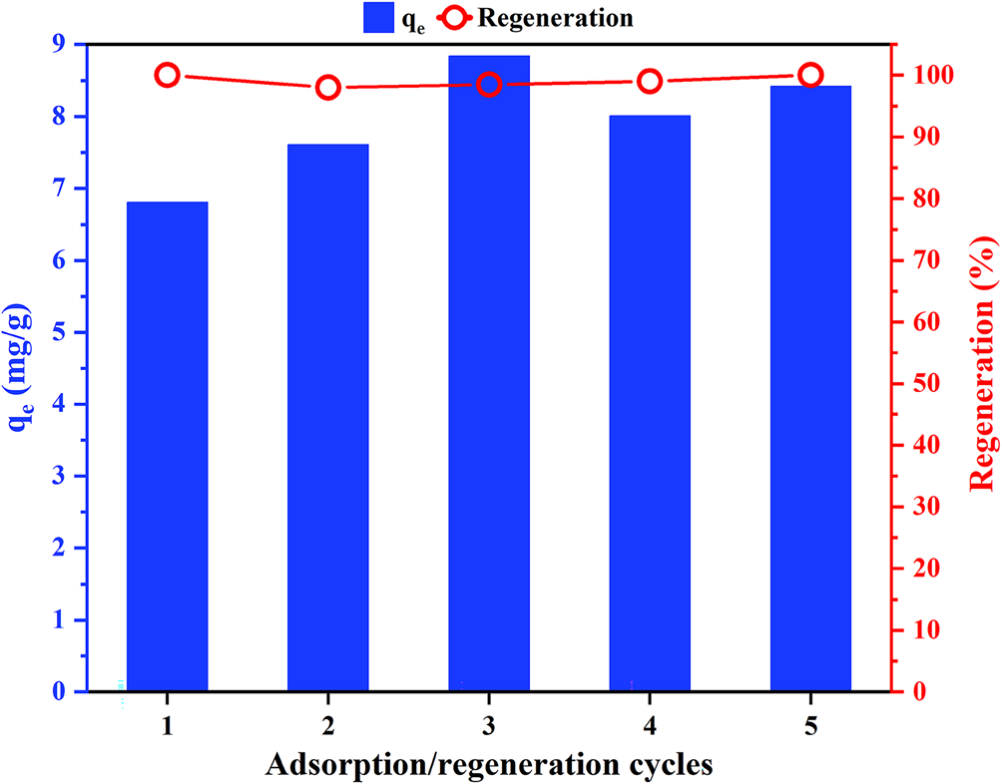
Adsorption/regeneration cycles of Oxi-CC for BTR adsorption.

The reusability is one of the critical features that determine the feasibility of practical application of an adsorbent^64^. A reusable adsorbent reduces both the cost of the adsorption process and the generated solid waste^65^. The primary requirement of a reusable adsorbent is regenerability. In the regeneration step the adsorption sites are freed-up from the occupying adsorbate molecules thus restoring the adsorption capacity. It is argued that only water can regenerate an adsorbent when the adsorption process is physisorption^66^. Therefore, based on the results of the kinetic and isotherm studies, deionized water was selected as the eluent in this study. Figure 5 shows that the adsorbed BTR can be nearly completely desorbed from the Oxi-CC using deionized water as the regeneration efficiency ranged between 98 and 100%. Also, it can be observed that the Oxi-CC retained it adsorption capacity after five cycles. The adsorption capacity in the first cycle was 6.81 mg/g and increased in the range 7.61 mg/g to 8.84 mg/g from the second to the fifth cycle. Previous researchers^65,67^ reported an increase in the adsorption capacity of other adsorbents relative to that of the first cycle.

## Conclusions

Carbon cloth has several attracting characteristics that encourage its application in water/wastewater treatment. Enhancing the adsorption characteristics of the carbon cloth and understating the adsorption mechanism is required to enable its practical application. In this study, HNO_3_ acid oxidation of a commercial carbon cloth introduced oxygen-containing functional groups and increased the surface area. Enhancing these features lead to improving the adsorption affinity toward benzotriazole. The adsorption of benzotriazole onto the oxidized carbon cloth was fast and occurred via surface adsorption at pH 9. Increasing the ionic strength of the benzotriazole solution improved the adsorption efficiency, and proved the involvement of like-charge and non-electrostatic attraction mechanisms. The pseudo-first-order model was the most compatible with the adsorption kinetics data. While the adsorption isotherm data was predicted efficiently by Freundlich, D–R, and Sips models. The regeneration and reuse study revealed that the Oxi-CC can be easily generated using deionized water and can be reused for five cycles without loss of adsorption capacity. Overall, the kinetic, isotherm, and regeneration studies indicated that BTR adsorbs perpendicularly onto the heterogeneous surface of Oxi-CC via physical interactions involving Van der Waal’s forces and forms multilayer. Conclusively, oxidized carbon cloth has great potential for practical application in water/wastewater treatment by the virtue of its high adsorption efficiency, ease of separation post-treatment, ease of regeneration, and reusability.

### Supplementary Information

Supplementary Information.

## Data Availability

All data generated or analyzed during this study are included in this published article and its Supplementary Information file.
