# Supplementary material for: Rapid adsorption of benzotriazole onto oxidized carbon cloth as an easily separable adsorbent
Source: Sci Rep. 2023 Oct 9;13:17030. doi: 10.1038/s41598-023-44067-w (PMC10562377; doi:10.1038/s41598-023-44067-w)
Supplement: Supplementary file 1 — Supplementary Information. [file 41598_2023_44067_MOESM1_ESM.pdf]

# Supporting Information

## Rapid adsorption of benzotriazole onto oxidized carbon cloth as an easily separable adsorbent

Emad K. Radwan [1*], Rehab A. Omar [1] & Ahmed S. Moursy [1]

[1] Water Pollution Research Department, National Research Centre, 33 El Buhouth St, Dokki, 12622 Giza, Egypt.

## Contents

**Kinetics and isotherm models.** ........ S2

Table S1. Non-linear forms of the applied kinetics and isotherm models. ........ S2

**Regeneration efficiency** ........ S3

**Figures** ........ S4

Figure S1. (a) UV spectrum and (b) calibration curve of benzotriazole. ........ S4

**References** ........ S5

* Corresponding author. Tel. +202 33370931; Fax: +202 33371211. E-mail emadk80@gmail.com; ek.hafez@nrc.sci.eg.

**Kinetics and isotherm models.**

The kinetic data was fitted to the pseudo-first-order (PFO) [1], pseudo-second-order (PSO) [2], and Elovich [3] models. While the isotherm data were fitted to Freundlich [4], Langmuir [5], Dubinin–Radushkevich (D–R) [6], and Sips [7] models. Table S1 gives the non-linear forms of the models and their parameters.

Table S1. Non-linear forms of the applied kinetics and isotherm models.

| Model | Non-linear form | Parameter |
|---|---|---|
| | **Kinetic models** | |
| **PFO** | $q_t = q_e\,(1 - e^{-k_1 t})$ | $q_t$ and $q_e$ (mg/g) are the amount of BTR adsorbed per gram of carbon cloth at equilibrium and contact time $t$ (min), respectively, $k_1$ (1/min) is the rate constant. |
| **PSO** | $q_t = \frac{k_2 q_e^2 t}{1 + k_2 q_e t}$ | $k_2$ (g/mg min) is the rate constant. |
| **Elovich** | $q_t = \frac{1}{\beta}\ln(1 + \alpha\beta t)$ | $\alpha$ (mg/(g min)) is the initial adsorption rate of and $\beta$ (g/mg) is constant related to the surface coverage and activation energy. |
| | **Isotherm models** | |
| **Freundlich** | $q_e = K_F\, C_e^{1/n}$ | $k_F$ (mmol$^{(1-1/n)}$L$^{(1/n)}$/g) and $n$ (–) are constants. |
| **Langmuir** | $q_e = \frac{q_L K_L C_e}{1 + K_L C_e}$ | $q_L$ (mmol/g) is the monolayer saturation capacity, and $k_L$ (L/mmol) is the equilibrium constant. |
| **D-R** | $q_e = q_{D\text{-}R}\, e^{\left[-K_{D\text{-}R}\left(RT\ln\left(1+\frac{1}{C_e}\right)\right)^2\right]}$ | $q_{D–R}$ (mmol/g) is the maximum adsorption capacity, $K_{D–R}$ (kmol$^2$/J$^2$) is a constant related to the mean free energy of adsorption ($E$, kJ/mol), R is the universal gas constant (kJ/mol.K), and T (K) is the absolute temperature. |
| **Sips** | $q_e = \frac{q_S\, K_S\, C_e^{n_S}}{1 + K_S\, C_e^{n_S}}$ | $q_S$ (mmol/g) is the maximum adsorption capacity, $K_S$ (L/mmol) is equilibrium constant, and $n_S$ (–) is an exponent. |

The degree of fitting the kinetic and isotherm models to the experimental results was judged and compared based on the values of the coefficient of determination ($R^2$, Eq. S1), chi-square ($\chi^2$, Eq. S2), and root mean square error (RMSE, Eq. S3). Higher $R^2$ value and lower $\chi^2$ and RMSE values indicate better fit.

$$R^2 = \frac{\sum \left(q_{e,cal} - \bar{q}_{e,exp}\right)^2}{\sum \left(q_{e,cal} - \bar{q}_{e,exp}\right)^2 - \sum \left(q_{e,cal} - q_{e,exp}\right)^2} \quad \text{(S1)}$$

$$\chi^2 = \sum_{i=1}^{N} \left[\frac{\left(q_{e,exp} - q_{e,cal}\right)^2}{q_{e,cal}}\right] \quad \text{(S2)}$$

$$RMSE = \sqrt{\frac{1}{N-M}\sum_{i=1}^{N}\left(q_{e,exp} - q_{e,cal}\right)^2} \quad \text{(S3)}$$

where $q_{e,exp}$ and $q_{e,cal}$ are the experimental and model calculated values of $q_e$, respectively, N is the number of data points, $\bar{q}_{e,exp}$ is the average of $q_{e,exp}$, and M is the number of model's variables.

**Regeneration efficiency**

The regeneration efficiency was calculated after each desorption cycle according to the following equations.

$$q_{desorbed} = \left(\frac{C_{eluent}}{m_{exhausted}}\right) \times V_{eluent} \quad \text{(S4)}$$

$$\text{Regeneration efficiency} = \left(\frac{q_{desorbed}}{q_{adsorbed}}\right) \times 100 \quad \text{(S5)}$$

where $q_{desorbed}$ and $q_{adesorbed}$ are the amount (mg/g) of desorbed and adsorbed BTR, respectively, $m_{exhausted}$ is the weight (mg) of the exhausted Oxi-CC, $V_{eluent}$ is the volume (mL) of deionized water used as an eluent, and $C_{eluent}$ is the concentration (mg/L) of BTR in the eluent.

**Figures**

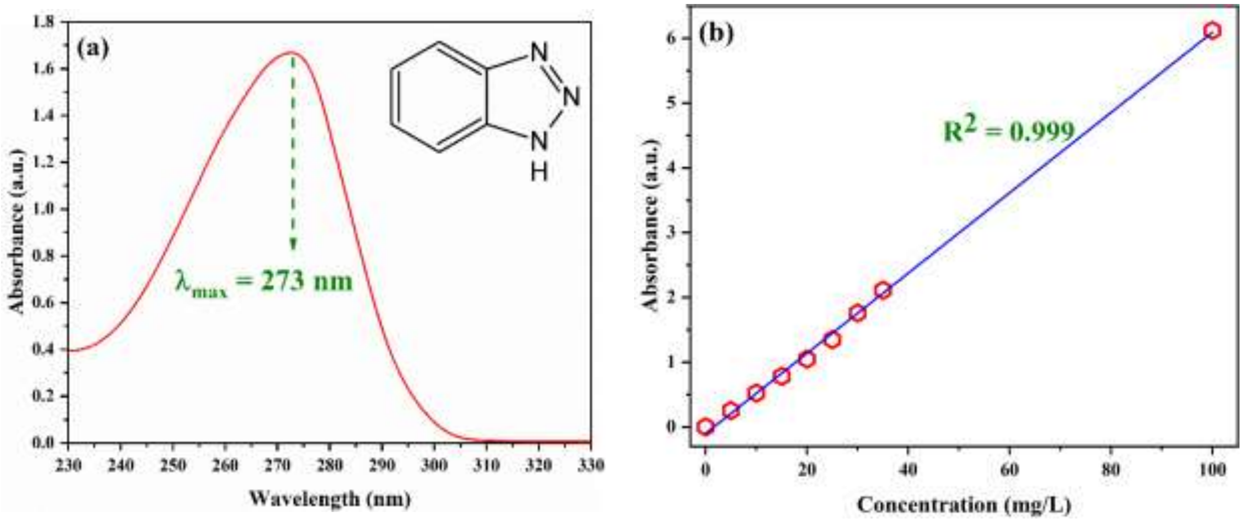


Figure S1. (a) UV spectrum and (b) calibration curve of benzotriazole.

## References


1 Langergren, S. & Svenska, B. K. Zur theorie der sogenannten adsorption geloester stoffe. *Veternskapsakad Handlingar* **24**, 1-39 (1898).

2 Blanchard, G., Maunaye, M. & Martin, G. Removal of heavy metals from waters by means of natural zeolites. *Water Research* **18**, 1501-1507, doi:https://doi.org/10.1016/0043-1354(84)90124-6 (1984).

3 Roginsky, S. & Zeldovich, Y. B. The catalytic oxidation of carbon monoxide on manganese dioxide. *Acta Phys. Chem. USSR* **1**, 2019 (1934).

4 Freundlich, H. M. F. Over the adsorption in solution. *Journal of Physical Chemistry* **57**, 385-470 (1906).

5 Langmuir, I. The adsorption of gases on plane surfaces of glass, mica and platinum. *Journal of the American Chemical society* **40**, 1361-1403 (1918).

6 Dubinin, M. M. & Radushkevich, L. V. The equation of the characteristic curve of the activated charcoal. *Proc. Acad. Sci. USSR Phys. Chem. Sect.* **55**, 331-337 (1947).

7 Sips, R. Combined form of Langmuir and Freundlich equations. *J. Chem. Phys* **16**, 490-495 (1948).
